# Genetic correlations of alcohol consumption and alcohol use disorder with sex hormone levels in females and males

**DOI:** 10.21203/rs.3.rs-3944066/v1

**Published:** 2024-02-28

**Authors:** Cameron Waller, Ada Ho, Anthony Batzler, Jennifer Geske, Victor Karpyak, Joanna Biernacka, Stacey Winham

**Affiliations:** Mayo Clinic; Mayo Clinic; Mayo Clinic; Mayo Clinic; Mayo Clinic; Mayo Clinic; Mayo Clinic

**Keywords:** alcohol consumption, alcohol use disorder, testosterone, estradiol, SHBG, genetic correlation

## Abstract

**Background:**

Alcohol consumption behaviors and alcohol use disorder risk and presentation differ by sex, and these complex traits are associated with blood concentrations of the steroid sex hormones, testosterone and estradiol, and their regulatory binding proteins, sex hormone binding globulin (SHBG) and albumin. Genetic variation is associated with alcohol consumption and alcohol use disorder, as well as levels of steroid sex hormones and their binding proteins.

**Methods:**

To assess the contribution of genetic factors to previously described phenotypic associations between alcohol-use traits and sex-hormone levels, we estimated genetic correlations (r_g_) using summary statistics from prior published, large sample size genome-wide association studies (GWAS) of alcohol consumption, alcohol dependence, testosterone, estradiol, SHBG, and albumin.

**Results:**

For alcohol consumption, we observed positive genetic correlation (i.e. genetic effects in the same direction) with total testosterone in males (r_g_ = 0.084, p = 0.007) and trends toward positive genetic correlation with bioavailable testosterone (r_g_ = 0.060, p = 0.084) and SHBG in males (r_g_ = 0.056, p = 0.086) and with albumin in a sex-combined cohort (r_g_ = 0.082, p = 0.015); however in females, we observed positive genetic correlation with SHBG (r_g_ = 0.089, p = 0.004) and a trend toward negative genetic correlation (i.e. genetic effects in opposite directions) with bioavailable testosterone (r_g_ = −0.064, p = 0.032). For alcohol dependence, we observed a trend toward negative genetic correlation with total testosterone in females (r_g_ = −0.106, p = 0.024) and positive genetic correlation with BMI-adjusted SHBG in males (r_g_ = 0.119, p = 0.017). Several of these genetic correlations differed between females and males and were not in the same direction as the corresponding phenotypic associations.

**Conclusions:**

Findings suggest that shared genetic effects may contribute to positive associations of alcohol consumption with albumin in both sexes, as well as positive associations between alcohol consumption and bioavailable testosterone and between alcohol dependence and SHBG in males. However, relative contributions of heritable and environmental factors to associations between alcohol-use traits and sex-hormone levels may differ by sex, with genetic factors contributing more in males and environmental factors contributing more in females

## Introduction

Alcohol use disorders have high prevalence that differs by sex and or by gender in many populations around the world^1–4^. Physiological differences by biological sex include both the pharmacokinetics and pharmacodynamics (physiological response) of acute and chronic exposure to ethanol^5,6^. Acute intoxication presents differently in females and males, and even at lesser doses of ethanol proportional to body mass, females have greater susceptibility to organ damage and other chronic health problems, such as hepatitis, hypertension, cardiomyopathy, diabetes, peripheral neuropathy, and volumetric brain loss^3,7^ These sex differences might relate to the genomic composition of sex chromosomes in females (XX) and males (XY) and to endogenous concentrations of sex hormones^8^, which are the major focus of this study. Furthermore, it is important to recognize that these physiological sex differences also combine with the complex social, cultural, and other environmental aspects of gender that might influence alcohol consumption behaviors (e.g. when, where, with whom, which types, and how much) and to the onset, presentation, likelihood of seeking help, and recovery from addiction^3^. Knowledge of the complex interactions between sex and gender and how these factors contribute to alcohol use behaviors and alcohol use disorders could inform preventive lifestyle decisions and actionable clinical interventions that improve patient prognosis.

Endogenous concentrations of the steroid sex hormones differ between females and males, and these hormones regulate many aspects of physiology. Though the main synthesis of testosterone and estradiol occurs within the gonads, their regulatory influence extends far beyond the primary and secondary sex organs. Hence it is essential to maintain proper balance in the bioactivity of these hormones, and sequestration by the binding proteins sex hormone binding globulin (SHBG)^9^ and albumin modulate the transport of hormones through the bloodstream and their accessibility to their respective receptors^10–12^. The synthesis of both SHBG and albumin occurs primarily in the liver, which also bears the burden of eliminating most ethanol from the bloodstream^13^; therefore, the liver’s function and susceptibility to damage constitutes one direct physiological link between alcohol use and the regulation of sex-hormone bioactivity.

Evidence from multiple prior studies demonstrated that the concentrations of steroid sex hormones and their binding proteins share sex-specific associations with the distinct traits of alcohol consumption and alcohol use disorder. Alcohol consumption is positively associated with levels of bioavailable testosterone^14–17^ and albumin^14,18^ in both sexes, but positive associations with total testosterone^14–17,19^ and estradiol^14–17,19,20^ have been observed consistently in females only. Similarly, alcohol use disorder is positively associated with total testosterone^14,19^ in both males and females, but associations with bioavailable testosterone^14^ and estradiol^14,19,21^ differ by sex and by menopause status in females. While alcohol consumption is positively associated with SHBG^15,20^ in premenopause (luteal phase) females, it is negatively associated with SHBG^14–17^ in postmenopause females and in males. In contrast, alcohol use disorder in both females and males is positively associated with SHBG^14,21^ and negatively associated with albumin^14^. These complex associations suggest that steroid sex hormones and their binding proteins (especially SHBG) may have an intimate connection with sex differences in alcohol use.

Beyond endogenous concentrations of sex hormones, genetics are another major contributor to the physiological differences of biological sex^8^. Sex chromosome complement differs between females (XX) and males (XY), and several proteins with pervasive influence are encoded by genes on these sex chromosomes, such as prominent epigenetic and protein regulatory genes KDM6A, KDM5C, and OGT^8^, as well as the gene encoding the androgen receptor (AR)^22^. Sex-specific concentrations of bioavailable steroid sex hormones bind and activate the AR or the estrogen receptors (ERalpha, ERbeta), which function in part as ligand-activated, nuclear-receptor transcription factors that regulate the expression of many genes. In fact, there are differences by sex in the transcriptional expression of roughly a third of all human genes across the genome, including the autosomes^23^. Furthermore, while levels of sex hormones are highly heritable in both females and males^24^, multiple recent studies have determined that even the autosomal (chromosomes 1–22) genetic etiologies of testosterone, estradiol, and SHBG differ profoundly by sex^25–28^. As an estimate of shared genetic effects across common autosomal SNPs, the genetic correlation between females and males is near 0% for testosterone^26–28^, about 7% for estradiol^25,28^, and approximately 83% for SHBG^26–28^.

Genetics also contribute to predisposition for alcohol-use traits, with heritability estimates of about 49% for alcohol use disorder^29,30^. Multiple genome-wide association studies (GWAS) have described the distinct genetic etiologies both for alcohol use behaviors (including frequency and quantity of alcohol consumption) and alcohol use disorders (including severe alcohol dependence)^31–41^; however, it is not yet known whether the genetic etiologies of these traits differ by sex^6^. Previous GWAS on both alcohol consumption and alcohol use disorder in sex-stratified cohorts found no difference in the SNP heritability by sex, and the genetic correlation between females and males was not significantly less than one^36,40–42^. Similar analyses using sex-stratified GWAS for alcohol dependence were inconclusive, partly due to limitations of sample size in the female cohort^38,42^. While there is currently limited evidence of direct differences in the genetic etiologies of alcohol use traits between females and males^6^, there are some similarities between the heritable predispositions of alcohol-use traits and sex hormone-related traits of female physiology. In females, greater alcohol consumption quantity and problematic alcohol use are genetically correlated with older age at menarche^31,34,36,37,40^, younger age at menopause^33,35^, and reduced likelihood of bilateral oophorectomy^36^. These alcohol-use traits are also genetically correlated with the use of oral contraception at a younger age^31,32,35,36^ and the lifetime use of hormonal therapy^31,32,35^. Genetic correlation between these hormone-related traits in females, which may serve as surrogate markers of hormone levels, and alcohol use suggests that genetic factors might underlie some of the associations between alcohol-use and sex-hormone phenotypes.

Given the heritability of alcohol use behaviors, alcohol use disorders, and levels of steroid sex hormones and their binding proteins, coupled with the suggestive genetic relationships of alcohol use with multiple surrogate markers of sex hormone levels in females, it is likely that both heritable and environmental factors contribute to observed associations between alcohol-use and sex-hormone phenotypes. We hypothesized that alcohol-use traits share some aspects of their genetic etiology with the heritable contributions to levels of steroid sex hormones and their binding proteins. We designed this study to quantify the broad, autosome-wide genetic similarity or dissimilarity between these traits. By utilizing publicly available summary statistics from previously published, large sample size genome-wide associations studies (GWAS) on alcohol-use and sex-hormone traits within large cohorts, we estimated genetic correlations between these traits and compared them to the previously published associations between corresponding phenotypes.

## Methods

### Summary statistics for large-cohort GWAS on alcohol use and sex hormones

We utilized summary statistics from previous large sample size genome-wide association studies (GWAS) of alcohol-use traits and blood concentrations of the steroid sex hormones to estimate the shared genetic etiology between these traits. Supplementary Table S1 provides details about the summary statistics that we used from prior GWAS on quantity of alcohol consumption^37^, risk of alcohol dependence^38^, and blood concentrations of total testosterone^28^, bioavailable testosterone^28^, estradiol^25^, SHBG^28^, and albumin^43^, including descriptions of the trait of interest, cohort sex, cohort ancestry, and sample size for each study (range N = 46,568 to N = 357,968). In some situations, the publicly available versions of these GWAS summary statistics that were most appropriate for estimates of genetic correlation differed slightly from the primary results reported in the respective publications (see Supplementary Methods). All sets of summary statistics included in our study came from GWAS or meta-analyses on cohorts of European ancestry. The GWAS on total testosterone^28^, bioavailable testosterone^28^, estradiol^25^, and SHBG^28^ used sex-stratified cohorts to accommodate major differences by sex in the physiological concentrations of these biomolecules as well as previously described differences by sex in their genetic etiologies^25–28^. In contrast, the GWAS on albumin^43^ used a sex-combined cohort as this protein does not demonstrate major differences in concentration or genetic etiology between females and males^27^. The GWAS on alcohol consumption quantity^37^ and alcohol dependence^38^ used sex-combined cohorts to maximize sample size, given that there is high genetic correlation between females and males in these traits.

### Estimates of SNP heritability and genetic correlation

To assess the shared genetic etiologies of alcohol-use traits and sex-hormone levels, we estimated SNP heritabilities (h^2^) and genetic correlations (r_g_) using Linkage Disequilibrium (LD) Score Regression (LDSC)^44,45^. From the summary statistics of the respective GWAS, LDSC accounts for LD between groups of SNPs (LD blocks) while estimating the SNP heritability^45^ of one individual trait or the genetic correlation^44^ between a pair of traits. The SNP heritability represents the proportion of variance in one trait that is attributable to variation across common genotype SNPs, which is a fraction of the total heritability (the proportion of phenotype variance due to all genetic factors)^46,47^. In contrast, the genetic correlation between two traits is a broad measure of the similarity (r_g_ > 0) or dissimilarity (r_g_ < 0) in the directionality and magnitude of their respective allelic effects (beta coefficients) across common genomic variants (SNPs) estimated in GWAS.

In this study, we estimated genetic correlations between pairwise combinations of alcohol-use traits (alcohol consumption and alcohol dependence) and sex-hormone levels (total testosterone, bioavailable testosterone, estradiol, SHBG, and albumin). Comparison of these genetic correlations to the corresponding phenotypic associations previously reported between alcohol-use traits and sex-hormone levels offers insight about the contribution of genetic factors to the associations observed directly between these phenotypes. Where the direction of significant genetic correlation (r_g_ < 0; r_g_ > 0) between two traits matches the direction of association between corresponding phenotypes (beta < 0; beta > 0), the shared genetic effects might contribute to the observed association. In contrast, if genetic correlation is in the opposite direction of a phenotypic association, non-genetic or environmental factors might exert a stronger influence on the association between phenotypes than the shared genetic effects. Similarly, where genetic correlation exists (r_g_ < 0; r_g_ > 0) without any association between corresponding phenotypes (beta = 0), environmental factors might oppose and mask the influence of genetic factors. Finally, a null genetic correlation (r_g_ = 0) suggests that there is not substantial, broad overlap in the genetic etiologies of two traits and that the association between phenotypes might relate most to non-genetic, environmental factors.

### Statistical comparisons and correction for multiple testing

To adjust for multiple testing, we considered the number of independent comparisons. Alcohol consumption and alcohol dependence are highly correlated, as are levels of the steroid sex hormones; in fact, bioavailable testosterone is a formulaic estimate from the actual concentrations of total testosterone, SHBG, and albumin^11^ such that concentrations of these biomolecules are completely dependent. For all hormones and proteins except for albumin, we conducted analyses with stratification by sex to accommodate biologically relevant differences. Due to the correlation among variables in this study, direct application of Bonferroni correction for the total number of comparisons is not appropriate here. Instead, we reported uncorrected p-values and, considering the extensive non-independence between these biomolecules and the alcohol-use traits, we recommend a threshold of p < 0.01 for statistical significance, and we report 99% confidence intervals for all estimates of genetic correlation.

## Results

To evaluate the extent to which shared genetic effects correspond to the previously reported associations between alcohol-use and sex-hormone traits, we estimated genetic correlations between prior-published GWAS summary statistics for the respective traits. Table S1 presents estimates of SNP heritability (h^2^) and details about the same sets of GWAS summary statistics that we used for each trait (see Supplementary Results). In Table 1, we present genetic correlations (r_g_) of alcohol consumption^37^ with the steroid sex hormones and their binding proteins, and in Table 2 we similarly present genetic correlations with alcohol dependence^38^. For convenient comparison with these novel genetic correlations in Tables 1 and 2, we also present adjacently the phenotypic associations of the same alcohol-use traits with levels of the steroid sex hormones and their binding proteins from a recently published study on data from the UK Biobank^14^.

When estimating genetic correlations of alcohol consumption with testosterone, estradiol, SHBG, and albumin, we found that alcohol consumption was positively genetically correlated with total testosterone in males (r_g_ = 0.0837, p = 0.0068), with a similar slight positive trend for bioavailable testosterone in males (r_g_ = 0.0591, p = 0.0844) (Table 1). In females, alcohol consumption was not genetically correlated with total testosterone (r_g_ = 0.0132, p = 0.6704) but trended toward negative genetic correlation with bioavailable testosterone (r_g_ = −0.0642, p = 0.0317) (Table 1). There was no significant genetic correlation of alcohol consumption with estradiol in females (r_g_ = −0.1099, p = 0.3427) or males (r_g_ = −0.0094, p = 0.8852) (Table 1), although standard errors were large and confidence intervals were wide. Alcohol consumption was positively genetically correlated with SHBG in females (r_g_ = 0.0894, p = 0.0037) and trended weakly toward positive genetic correlation with SHBG in males (r_g_ = 0.0560, p = 0.0859) (Table 1); however, there was no genetic correlation when the GWAS of SHBG adjusted for BMI in females (r_g_ = 0.0271, p = 0.4013) and males (r_g_ = 0.0343, p = 0.3176) (Table 1). Alcohol consumption also trended toward positive genetic correlation with albumin in both sexes combined (r_g_ = 0.0819, p = 0.0150) (Table 1).

When estimating genetic correlations of alcohol dependence with the steroid sex hormones and their binding proteins, we found that alcohol dependence trended toward negative genetic correlation with total testosterone in females (r_g_ = −0.1058, p = 0.0243) but was not genetically correlated with total testosterone in males (r_g_ = 0.0420, p = 0.4133) or with bioavailable testosterone in either sex (females: r_g_ = −0.0528, p = 0.3105; males: r_g_ = −0.0362, p = 0.5230) (Table 2). There was no significant genetic correlation of alcohol dependence with estradiol in females (r_g_ = −0.1827, p = 0.3397) or males (r_g_ = 0.1594, p = 0.1849) (Table 2), although these estimates had large standard errors and wide confidence intervals. There was no genetic correlation of alcohol dependence with SHBG in females (r_g_ = −0.0206, p = 0.7006) or males (r_g_ = 0.0670, p = 0.1802); however, alcohol dependence trended toward positive genetic correlation with BMI-adjusted SHBG in males (r_g_ = 0.1185, p = 0.0174) but not in females (r_g_ = 0.0258, p = 6128) (Table 2). Alcohol dependence was not genetically correlated with albumin in males and females (r_g_ = 0.0054, p = 0.9110) (Table 2).

## Discussion

Our objective in this study was to assess the impact of broadly shared genetic etiologies on the relationship between alcohol-use traits and sex-hormone levels. Multiple previous studies have described associations of acute or chronic alcohol use behaviors (including frequency and quantity of consumption) and alcohol use disorders (including dependence) with blood concentrations of steroid sex hormones and their regulatory binding proteins^14–21^. Recently we described associations of alcohol consumption and alcohol dependence with testosterone, estradiol, SHBG, and albumin in data from the UK Biobank, with striking differences by sex^14^. In this study, we hypothesized that both environmental and heritable factors contribute to these associations between phenotypes, and to test this hypothesis, we estimated the genetic correlations of alcohol-use traits with sex-hormone levels using prior GWAS results. In males we observed positive genetic correlation of alcohol consumption with total testosterone and a slight positive trend with bioavailable testosterone, while in females we observed a trend toward negative genetic correlation of alcohol consumption with bioavailable testosterone and a trend toward negative genetic correlation of alcohol dependence with total testosterone. SHBG was positively genetically correlated with alcohol consumption in females and trended slightly toward positive genetic correlation with alcohol consumption in males. SHBG also trended toward positive genetic correlation with alcohol dependence in males, but only after adjustment for BMI in the GWAS of SHBG. We also observed a trend toward positive genetic correlation between alcohol consumption and albumin in both sexes. Collectively, these findings may suggest that sex-specific heritable predispositions for higher or lower levels of steroid sex hormones and their binding proteins contribute to alcohol-use behaviors and disorders in some contexts while environmental factors dominate in others^48^.

When compared to previously reported associations between hormone levels and alcohol consumption and dependence, we observed three instances in which the direction of genetic correlation was consistent with the corresponding association between phenotypes^14^. The positive genetic correlations of alcohol consumption with bioavailable testosterone in males and with albumin in a sex-combined cohort paralleled the previously published positive phenotypic associations. Additionally, alcohol dependence was positively associated with BMI-adjusted levels of SHBG and was also positively genetically correlated with BMI-adjusted SHBG in males. In these instances, the shared genetic effects might contribute to the positive associations observed between phenotypes.

However, other genetic correlations of alcohol traits with steroid sex hormones and their binding proteins were inconsistent with the corresponding associations between phenotypes^14^. Specifically, alcohol consumption was positively genetically correlated with total testosterone in males but not in females, even though the corresponding phenotypes were positively associated in females but not in males. While alcohol consumption associated positively with bioavailable testosterone levels in both sexes, the corresponding genetic correlation was negative in females unlike males. Alcohol consumption was not genetically correlated with estradiol in either sex, even though the corresponding phenotypes associated positively in females. Levels of BMI-adjusted SHBG were negatively associated with alcohol consumption in both sexes, but the only genetic correlations in either sex were positive and without adjustment for BMI.

In contrast to alcohol consumption, alcohol dependence shared a different pattern of phenotypic associations^14^ and genetic correlations with steroid sex hormones and their binding proteins. Total testosterone was negatively genetically correlated with alcohol dependence in females but not in males, despite positive associations between the corresponding phenotypes in both sexes. While alcohol dependence was positively associated with levels of bioavailable testosterone in females and negatively associated in males, there were no corresponding genetic correlations. Similarly, alcohol dependence and estradiol were not genetically correlated in either sex, even though there was a positive association between corresponding phenotypes in males. Levels of BMI-adjusted SHBG were positively associated with alcohol dependence in both sexes, but there was no genetic correlation in females unlike males. Levels of albumin were negatively associated with alcohol dependence but did not have any corresponding genetic correlation.

Overall, we found that most of the prior published associations^14^ between alcohol-use traits and sex-hormone levels did not correspond to genetic correlations with consistent directionality, suggesting that heritable and environmental factors related to alcohol use and sex hormones may be complex and conflicting. While it is important to acknowledge that null estimates of genetic correlation (a broad, autosome-wide measure) cannot dismiss the possibility of narrower, region-specific pleiotropy, the instances of genetic correlation in the absence of phenotypic association or in an opposite direction suggest that complex environmental factors might mask or even overpower the influence of any shared heritable factors between some of these alcohol-use and sex-hormone traits. Our results also suggest that the relative contributions of heritable and environmental factors to the relationships between alcohol-related traits and sex-hormone levels are likely different between males and females. Notably, the three instances in which traits shared both positive phenotypic associations and positive genetic correlations corresponded to male-only or sex-combined cohorts, whereas there were no instances of genetic correlation consistent with corresponding associations between alcohol-related traits and sex hormone levels in females. Furthermore, as was previously seen when comparing hormone levels with alcohol consumption and dependence, patterns of genetic correlation were also different between males and females, with significant genetic correlation observed in one sex but not the other or even trends toward genetic correlation in opposite directions in either sex. Collectively, the observed genetic correlations and lack of direction consistent with phenotype associations in females suggest that the phenotypic associations between these alcohol-use traits and sex-hormone levels may relate more to environment in females and more to shared heritability in males. The sex-specific genetic etiologies^25–28^ of testosterone, estradiol, and SHBG support the hypothesis that there are sex differences in the influences of heritable and environmental factors, although further investigation is necessary.

The example of SHBG illustrates the complexity of studying genetic etiology in the context of dynamic regulation and environmental factors. As described previously, we determined that SHBG moderated the associations of total testosterone with both alcohol consumption and alcohol use disorder in males only, whereas in both females and males, SHBG mediated these same associations^14^. Clearly this high-affinity binding protein with tightly regulated and dynamic synthesis in the alcohol-sensitive liver has an important role in the relationship between alcohol use and the steroid sex hormones, especially in males^14^ In this study, we observed major differences in the genetic correlation of SHBG with both alcohol consumption and alcohol dependence that depended on adjustment for BMI in the GWAS of SHBG. Notably, higher BMI is associated with lower concentrations of SHBG in the blood^49,50^; however, it is not general adiposity (subcutaneous or visceral) that drives this association^49^. Rather, obesity and excessive alcohol consumption both can cause damage to the liver^51^ in the form of fatty liver disease and steatohepatitis, and this fat accumulation and inflammation of the liver might in turn decrease synthesis of SHBG.^49^ As higher BMI is also associated with higher alcohol consumption^52^, BMI has the potential to impart collider bias^53^ on the association between SHBG and alcohol use; therefore, interpretation of the results from GWAS of SHBG with or without BMI adjustment is complex, as acknowledged by the authors who published these GWAS summary statistics.^28^ Thus, we chose to report genetic correlations for SHBG both with and without adjustment for BMI, even though the GWAS of SHBG adjusted for BMI showed greater SNP heritability^28^.

While the GWAS summary statistics that we used in this study provided many strengths including large sample sizes, some limitations also need to be acknowledged. First, there are inherent challenges to describing the genetic etiologies of the traits used in our study. Complex sociocultural and other environmental factors contribute to alcohol use behaviors^3^, and it is also difficult for cross-sectional, single-time-point measurements of steroid sex hormones and their binding proteins to represent adequately the dynamic concentrations that fluctuate within individual persons on diurnal, monthly, and seasonal rhythms with influence from many other factors, especially in females. Second, GWAS of sex- hormone levels may not have adjusted for all relevant covariates particularly in females. Specifically, the GWAS^28^ for total testosterone in females did not adjust for aspects of female physiology such as phase of the menstrual cycle, menopause, or use of oral contraception and other hormonal therapies. The GWAS in females for bioavailable testosterone^28^ and for estradiol^25^ adjusted for some of these covariates. Furthermore, the GWAS of testosterone and estradiol were not stratified by premenopause and postmenopause female stages of life, which correspond to major differences in the concentrations of these steroid sex hormones. Whereas previous studies have performed GWAS of testosterone and estradiol in female cohorts with stratification by premenopause and postmenopause stages of life^27,54^ the summary statistics are not publicly available to our knowledge. Third, the available GWAS summary statistics for steroid sex hormones and their binding proteins did not capture genetic etiologies earlier in the life course before age 40. These GWAS for hormone levels used data from the UK Biobank, which represents a more advanced stage of life^55^, with a youngest age at the time of enrollment and blood draw of approximately 40 years in both females and males^56^. Fourth, partly related to reasons above, estimates of SNP heritability and genetic correlation based on these GWAS summary statistics had varying degrees of precision. While the SNP heritabilities were high for total testosterone, bioavailable testosterone, SHBG, and albumin, SNP heritability estimates for estradiol were much lower with large standard errors. Notably, the biochemical assay used to measure concentrations of estradiol in UK Biobank samples lacked sensitivity (lower limit of detection)^57^, and investigators accommodated the high rates of missingness in these measurements by performing GWAS on a dichotomous variable for the detectability of estradiol^25–28^. Due to smaller effective sample size and the dichotomization of a continuous trait, there was lower precision in estimates of SNP heritability and genetic correlation for estradiol, which may have obscured any shared genetic architecture with alcohol-use traits.

This study assessed the shared genetic etiologies between alcohol-use traits and sex-hormone levels across the autosomes, which leaves an important knowledge gap for future investigation, given the relevance of chromosomes X and Y to sex differences of many traits. Historically, the majority of GWAS have ignored the sex chromosomes, and this unfortunate exclusion persists today. A recent review estimated that of all publicly available GWAS summary statistics, only 25% included variants on chromosome X and only 3% included variants on chromosome Y^58^. The sex chromosomes present unique features compared to the autosomes, including sex-dependent diploid or haploid dosages of X, recombination only within the pseudo-autosomal region of Y, and the developmental mosaic of X-inactivation in females^58^, which lead to several analytical challenges that are beginning to be addressed^59^. However, many analytical methods avoid these challenges by ignoring the sex chromosomes, including currently available methods to estimate SNP heritability and genetic correlation, such as LDSC^44,45^. The genetic etiologies of testosterone, estradiol, and SHBG all differ quantitatively between females and males even when only considering the autosomes^25–28^, but prior GWAS have demonstrated additional genome-wide-significant associations with variants on the X chromosome^25,28^. Furthermore, for the alcohol use traits, the recent meta-analysis GWAS on alcohol consumption and alcohol use disorder excluded the sex chromosomes^31,37,38^. Due to the exclusion of chromosomes X and Y, the estimates of SNP heritability reported here may be artificially low, and it is possible that the genetic etiologies of alcohol use and sex hormones share additional pleiotropy across variants on the sex chromosomes. Indeed, a previous study found that variants on the X chromosome contributed to sex differences in multiple complex heritable diseases^60^, and another study found evidence that sex-specific associations across autosomal variants alone were unlikely to explain the strong sex differences in several psychiatric traits^42^.

In conclusion, our study demonstrates evidence of shared effects of genetic variation between alcohol-use traits and sex-hormone levels, with differences between females and males. Patterns of genetic correlation of sex hormones with alcohol consumption differed from those with alcohol dependence, emphasizing the distinction between these alcohol-use traits. We found that shared genetic effects might contribute to the positive associations^14^ observed between alcohol consumption and levels of bioavailable testosterone in males, between alcohol consumption and levels of albumin in both sexes, and between alcohol dependence and levels of BMI-adjusted SHBG in males. We also found multiple instances of genetic correlations that differed by sex and did not correspond to phenotypic associations with consistent directionality, suggesting that relative contributions of heritable and environmental factors to the relationships between alcohol-use traits and sex-hormone levels may differ between females and males. The associations between alcohol-use traits and sex-hormone levels may relate more to environmental factors in females, while shared genetic factors may influence these associations more strongly in males. Future work should further evaluate this complex interaction of heritable and environmental^48^ factors, including sociocultural factors related to gender^3^. Additionally, some of the null genetic correlations that we observed could be false negative findings, and future analyses with newer methods^48,61,62^ that offer better resolution or better statistical power may demonstrate shared genetic effects or pleiotropy between these traits within narrower genomic regions or even specific genes. There is also a need for future GWAS on higher-quality measurements of sex-hormone levels in large cohorts, especially for estradiol, and these GWAS should stratify by sex, represent stages of life more inclusively, and adjust for important hormone-influencing factors, such as those specific to female physiology, including the menstrual cycle and menopause. Collectively, these future studies will be important to describe the relevance of steroid sex hormones and their binding proteins to differences by sex and gender in alcohol use behaviors and alcohol use disorders.

## Figures and Tables

**Table 1.** Genetic correlations and phenotypic associations of alcohol consumption with steroid sex hormones and their binding proteins

		Primary Trait: Alcohol Consumption						
		Genetic Correlation					Phenotype Association^1^	
Secondary Trait:	Sex	r_g_	se	99%ci low	99%ci high	p	beta	p

Testosterone, Total	Female	0.0132	0.0311	−0.0669	0.0933	0.6704	**0.019**	**< 0.001**
Testosterone, Total	Male	**0.0837**	**0.0309**	**0.0041**	**0.1633**	**0.0068**	−0.001	0.645

Testosterone, Bioavailable	Female	−**0.0642**	**0.0299**	−**0.1412**	**0.0128**	**0.0317**	**0.031**	**< 0.001**
Testosterone, Bioavailable	Male	**0.0591**	**0.0343**	−**0.0293**	**0.1475**	**0.0844**	**0.024**	**< 0.001**

Estradiol	Female	−0.1099	0.1158	−0.4082	0.1884	0.3427	**0.009**	**0.002**
Estradiol	Male	−0.0094	0.0650	−0.1768	0.1580	0.8852	−0.004	0.430

SHBG	Female	**0.0894**	**0.0308**	**0.0101**	**0.1687**	**0.0037**	NA	NA
SHBG	Male	**0.0560**	**0.0326**	−**0.0280**	**0.1400**	**0.0859**	NA	NA

SHBG-BMI	Female	0.0271	0.0323	−0.0561	0.1103	0.4013	−**0.023**	**< 0.001**
SHBG-BMI	Male	0.0343	0.0344	−0.0543	0.1229	0.3176	−**0.030**	**< 0.001**

Albumin	All	**0.0819**	**0.0337**	−**0.0049**	**0.1687**	**0.0150**		
Albumin	Female						**0.054**	**< 0.001**
Albumin	Male						**0.030**	**< 0.001**

We used prior published GWAS summary statistics and linkage disequilibrium score regression (LDSC) to estimate genetic correlations of alcohol consumption with steroid sex hormones and their binding proteins (Table S1), and we compared these novel genetic correlations to prior published associations between phenotypes corresponding to these traits. Table S1 presents further details on the respective sets of GWAS summary statistics that we used in this study. Effect coefficients (beta) from phenotypic associations are not on the same scale as genetic correlations (r_g_).

Abbreviations:

SHBG: sex hormone binding globulin; BMI: body mass index; SHBG-BMI: designation of GWAS on SHBG with adjustment for BMI as a covariate; GWAS: genome-wide association study; r_g_: genetic correlation; se: standard error; 99%ci low: lower limit of 99% confidence interval; 99%ci high: upper limit of 99% confidence interval; p: p-value for testing the null hypothesis that genetic correlation (r_g_) or phenotypic association (beta) is zero; NA: not available

1: Prior published phenotypic associations from Table 4 in Ho et al, 2023 (PubMed:36701934)

**Table 2.** Genetic correlations and phenotypic associations of alcohol dependence with steroid sex hormones and their binding proteins

		Primary Trait: Alcohol Dependence						
		Genetic Correlation					Phenotype Association^1^	
Secondary Trait:	Sex	r_g_	se	99%ci low	99%ci high	p	beta	p

Testosterone, Total	Female	−**0.1058**	**0.0470**	−**0.2269**	**0.0153**	**0.0243**	**0.211**	**< 0.001**
Testosterone, Total	Male	0.0420	0.0513	−0.0901	0.1741	0.4133	**0.225**	**< 0.001**

Testosterone, Bioavailable	Female	−0.0528	0.0521	−0.1870	0.0814	0.3105	**0.108**	**0.046**
Testosterone, Bioavailable	Male	−0.0362	0.0567	−0.1823	0.1099	0.5230	−**0.078**	**< 0.001**

Estradiol	Female	−0.1827	0.1913	−0.6755	0.3101	0.3397	−0.061	0.474
Estradiol	Male	0.1594	0.1203	−0.1505	0.4693	0.1849	**0.257**	**< 0.001**

SHBG	Female	−0.0206	0.0537	−0.1589	0.1177	0.7006	NA	NA
SHBG	Male	0.0670	0.0500	−0.0618	0.1958	0.1802	NA	NA

SHBG-BMI	Female	0.0258	0.0509	−0.1053	0.1569	0.6128	**0.124**	**0.002**
SHBG-BMI	Male	**0.1185**	**0.0498**	−**0.0098**	**0.2468**	**0.0174**	**0.430**	**< 0.001**

Albumin	All	0.0054	0.0484	−0.1193	0.1301	0.9110		
Albumin	Female						−**0196**	**< 0.001**
Albumin	Male						−**0.270**	**< 0.001**

We used prior published GWAS summary statistics and linkage disequilibrium score regression (LDSC) to estimate genetic correlations of alcohol dependence with steroid sex hormones and their binding proteins (Table S1), and we compared these novel genetic correlations to prior published associations between phenotypes corresponding to these traits. Table S1 presents further details on the respective sets of GWAS summary statistics that we used in this study. Effect coefficients (beta) from phenotypic associations are not on the same scale as genetic correlations (r_g_).

Abbreviations:

SHBG: sex hormone binding globulin; BMI: body mass index; SHBG-BMI: designation of GWAS on SHBG with adjustment for BMI as a covariate; GWAS: genome-wide association study; r_g_: genetic correlation; se: standard error; 99%ci low: lower limit of 99% confidence interval; 99%ci high: upper limit of 99% confidence interval; p: p-value for testing the null hypothesis that genetic correlation (r_g_) or phenotypic association (beta) is zero; NA: not available

1: Prior published phenotypic associations from Table 2 in Ho et al, 2023 (PubMed:36701934)

## Data Availability

This study applied prior published and publicly available computational software tools to process and analyze publicly available summary statistics from prior published genome-wide association studies (GWAS). Software tools and reference data are accessible from sources cited in the Methods and Supplemental Methods. Detailed information about the prior published GWAS is available from original publications cited in Table S1, and the summary statistics are accessible from online repositories including the Psychiatric Genomics Consortium (https://pgc.unc.edu/), the GWAS & Sequencing Consortium of Alcohol and Nicotine use (GSCAN) at the University of Minnesota (https://genome.psych.umn.edu/index.php/GSCAN), the National Human Genome Research Institute (NHGRI) and European Bioinformatics Institute (EBI) GWAS Catalog (https://www.ebi.ac.uk/gwas/), and the Zenodo archive (https://zenodo.org).
